# Development of a Luciferase Immunosorbent Assay for Detecting Crimean–Congo Hemorrhagic Fever Virus IgG Antibodies Based on Nucleoprotein

**DOI:** 10.3390/v17010032

**Published:** 2024-12-28

**Authors:** Qi Chen, Yuting Fang, Ning Zhang, Chengsong Wan

**Affiliations:** 1 Guangdong Provincial Key Laboratory of Tropical Disease Research, School of Public Health, Southern Medical University, Guangzhou 510515, China; chenqii@smu.edu.cn (Q.C.); 2450241012@mails.szu.edu.cn (Y.F.); znn@smu.edu.cn (N.Z.); 2 Guangdong Key Laboratory for Genome Stability & Disease Prevention, Shenzhen University School of Medicine, Shenzhen 518060, China

**Keywords:** Crimean–Congo hemorrhagic fever virus (CCHFV), nucleoprotein, luciferase immunosorbent assay (LISA), IgG, detection

## Abstract

Crimean–Congo hemorrhagic fever (CCHF) is a serious tick-borne disease with a wide geographical distribution. Classified as a level 4 biosecurity risk pathogen, CCHF can be transmitted cross-species due to its aerosol infectivity and ability to cause severe hemorrhagic fever outbreaks with high morbidity and mortality. However, current methods for detecting anti-CCHFV antibodies are limited. This study aimed to develop a novel luciferase immunosorbent assay (LISA) for the detection of CCHFV-specific IgG antibodies. We designed specific antigenic fragments of the nucleoprotein and evaluated their sensitivity and specificity in detecting IgG in serum samples from mice and horses. In addition, we compared the efficacy of our LISA to a commercial enzyme-linked immunosorbent assay (ELISA). Our results demonstrated that the optimal antigen for detecting anti-CCHFV IgG was located within the stalk cut-off domain of the nucleoprotein. The LISA exhibited high specificity for serum samples from indicated species and significantly higher sensitivity (at least 128 times) compared with the commercial ELISA. The proposed CCHFV-LISA has the potential to facilitate serological diagnosis and epidemiological investigation of CCHFV in natural foci, providing valuable technical support for surveillance and early warning of this disease.

## 1. Introduction

Crimean–Congo hemorrhagic fever (CCHF) is a tick-borne viral infection prevalent in over 50 countries across Asia, Africa, Southern Europe, and the Middle East [1,2]. Its epidemiological range is closely linked to the distribution of the Hyalomma tick, the predominant vector of the Crimean–Congo hemorrhagic fever virus (CCHFV) [3]. CCHFV-infected ticks can enter new continents through long-distance migration of birds, global trade, and other factors [4,5], leading to the continued expansion of the CCHFV geographic distribution. CCHF is characterized by its rapid onset of hyperthermia, gastrointestinal bleeding, thrombocytopenia, leukopenia, elevated liver enzymes, multi-organ failure, and shock, with a mortality rate that can exceed 30% in certain regions [6]. Iraq reported a new outbreak of CCHF in 2022, with at least 212 cases and 27 fatalities [7]. Recently, the resurgence of CCHF in Pakistan’s Balochistan province has resulted in 16 fatal cases, including a healthcare worker infected by hospitalized patients [8]. Given its high virulence, multiple modes of transmission, expanding geographic distribution, and significant threat to public health, the World Health Organization (WHO) has included CCHFV on the list of priority diseases for urgent research and development [9].

The CCHFV belongs to the genus Nairovirus in the Bunyaviridae family and was first reported in Crimea in 1945. Ticks carrying CCHFV, domestic animals, wildlife, and patients are all considered sources of CCHF [10]. In nature, the primary hosts of CCHFV are herbivorous vertebrates, which generally do not exhibit symptoms after infection but develop a transient viremia followed by the production of specific antibodies [11]. To date, over 20 species of vertebrates are known to be infected with CCHFV, including goats, sheep, hares, cattle, horses, and mice [12,13]. Although birds are generally less susceptible to CCHFV infection, recent studies in Senegal have found that certain non-finch birds that are regularly on the ground can develop low levels of viremia and a transient antibody response [14,15,16]. In addition, ostriches are capable of being infected by CCHFV, developing viremia, and being antibody-positive [17].

CCHFV particles are spherical or ellipsoidal and measure 80–100 nm in diameter. The CCHFV viral genome is a three-segmented negative-stranded RNA with small (S), medium (M), and large (L) segments encoding nucleoprotein (NP), glycoprotein precursor (GPC), and RNA-dependent RNA polymerase (RdRP), respectively [18]. The NP is the most abundant protein in the viral particle and is involved in various CCHFV biological processes. In addition, it is responsible for binding to viral genomic RNA to form the Ribonucleoprotein (RNP) complex that protects the genome from degradation by the host cell [19]. Moreover, it binds to transcriptional replication-associated proteins to initiate the transcription process [20]. Notably, it serves as a highly antigenic and immunogenic target for antiviral and clinical diagnostics due to its high levels in the early stages and being detected during the invasive phase of viral infection [18,21,22]. The stalk domain of NP, characterized by its cysteine-3 cleavage site, is the most accessible position in the entire molecule [23]. The strict conservation of this domain across all CCHFV strains suggests its suitability for detection purposes.

Rapid and convenient detection of CCHFV is key for effective prevention and control. Among them, serological tests play a crucial role in the surveillance and diagnosis of CCHFV infection. Specific antibodies can be detected to identify active or previous infections. Furthermore, seroprevalence studies allow for the assessment of population exposure to identify high-risk groups, as well as the monitoring of vaccine efficacy [24]. It has been reported that traditional enzyme-linked immunosorbent assays (ELISA) using full-length NP fragments could detect anti-CCHFV antibodies in sheep [25]. However, traditional serological analysis often requires the purification of monoclonal antibodies or antigens for detection, and the application of ELISAs can be constrained by species-specific enzyme-coupled antibodies [26,27]. Notably, it is often difficult to obtain generic specific antibodies from wildlife.

To address these limitations, we developed a novel cross-species detectable CCHFV IgG antibody method called luciferase immunosorbent assay (LISA). It allows for rapid and convenient detection of antibodies without the need for antigen purification or expression modification. LISA uses G protein to capture IgG antibodies in serum and a fusion protein of CCHFV NP and Nanoluc luciferase as the detection antigen. Importantly, this assay does not require the detection of a species-specific marker secondary antibody, thereby facilitating its broader utilization for timely monitoring and early warning of CCHF outbreaks and epidemics.

## 2. Materials and Methods

### 2.1. Cells and Serum Samples

HEK 239T cells were preserved in our laboratory and grown in 5% CO_2_ at 37 °C using Dulbecco’s Modified Eagle Medium (DMEM; Gibco, Waltham, MA, USA) supplemented with 10% fetal bovine serum (FBS; Gibco, Australia).

Serum samples from mice immunized with CCHFV NP (*n* = 29) and negative mice (*n* = 30) were prepared in our laboratory. Serum samples from CCHFV, Rift Valley Fever virus (RVFV), West Nile virus (WNV), Nipah virus (NiV), and Ebola virus (EBOV) positive horses were obtained from the Changchun Institute of Veterinary Medicine, Chinese Academy of Agricultural Sciences. Serum samples from patients infected with Chikungunya virus (CHIKV), Dengue virus (DENV), and hepatitis C virus (HCV), as well as normal human sera, were kept in our laboratory and validated in previous studies [28].

### 2.2. Construction of Recombinant Expression Plasmid

The DNA sequence encoding the NP of the CCHFV IbAr10200 strain (GenBank accession no. AAM48106) was subcloned into the pNLF1-N vector. Based on the structural features of NP [19], four amino acid sequences were designed, including the full-length NP (NP-full), the N-terminal head domain (NP-C1), the stalk domain (NP-C2), and the C-terminal head domain (NP-C3). We also added an HA-tag at the C-terminus to verify the correct expression of the fusion protein. PCR amplification was performed using pNLF1-N-NP-full as a template and primers from Table 1. The PCR products obtained were ligated into the linearized pNLF1-N vector and transformed into DH5α competent cells (Tsingke Biotech, Beijing, China), which were grown in Luria–Bertani medium with Ampicillin at 37 °C. Positive clones were identified by double enzyme digestion and DNA sequencing, and the primers in Table 1 were used for sequencing. The recombinant plasmids were extracted from the correctly sequenced strains after 16 h of oscillatory incubation at 37 °C.

### 2.3. Expression of Recombinant Luciferase Fusion Proteins

HEK 239T cells were seeded in 55 cm^2^ dishes (NEST, Wuxi, China) and transfected with 21 μg of plasmid DNA using Lipofectamine 3000 (Thermo Fisher Scientific, Waltham, MA, USA) following the manufacturer’s protocols. After 48 h, cells were washed twice with cold 0.01 M phosphate-buffered saline (PBS) and lysed on ice for 30 min with RIPA Lysis Buffer (Beyotime, Shanghai, China) and protease inhibitors (Solarbio, Beijing, China). Supernatants were collected after centrifugation at 15,000 rpm for 20 min, and the protein concentration of the lysate was determined using the BCA kit (Beyotime, Shanghai, China).

### 2.4. Western Blot

The cell lysate supernatant was added to the SDS-PAGE loading buffer and boiled for 10 min. SDS-PAGE was carried out according to the ratio of equal protein amounts, and the proteins were electrotransferred onto a polyvinylidene difluoride (PVDF) membrane (Millipore, Burlington, MA, USA). After blocking the membranes with 5% skimmed milk, the membranes were probed with rabbit anti-HA and α-tubulin antibodies (1:5000 dilution in TBST) at 4 °C overnight. Horseradish peroxidase (HRP)-conjugated goat anti-rabbit antibody (1:10000 dilution in TBST) was used as the secondary antibody. The blots were visualized using the ECL reagent according to the manufacturer’s instructions.

### 2.5. Preparation of Mice Serum

The CCHFV NP gene sequence was expressed and purified by Genscript (Nanjing, China). The NP gene sequence was cloned into the pET-30a (+) prokaryotic expression vector, and the protein was expressed using *E. coli*. The positive clone was inoculated into LB medium containing 50 μg/mL kanamycin in shaker at 37 °C with shaking at 200 rpm. When the OD600 reached about 1.2, cell culture was induced with 0.5 mM IPTG at 15 °C for 16 h. And the cells were harvested by centrifugation. Next, cell pellets were resuspended with lysis buffer followed by sonication. The precipitate after centrifugation was dissolved using denaturing agent. Target protein was obtained by two-step purification using Ni column+Q Sepharose column. Protein after refolding was sterilized by 0.22 μm filter before stored in aliquots. The purified protein was used as an immunogen and mixed with Freund’s complete adjuvant in equal proportions to prepare mock positive serum samples.

A total of 29 female BALB/c mice aged 6–8 weeks were immunized with 50 µg of the purified NP protein by subcutaneous injection. Booster immunizations were administered on days 14 and 28 following the initial immunization. After 1 week of the third infection, blood was collected from the posterior ocular venous plexus and centrifuged at 5000 rpm for 30 min to isolate the supernatant and obtain positive serum. Thirty mice were injected with the same volume of PBS to obtain negative serum under consistent separation conditions. The serums were stored at –80 °C for subsequent use in LISA development.

### 2.6. Development of CCHFV LISA Based on Different NP Fusion Proteins

**Figure 1:**
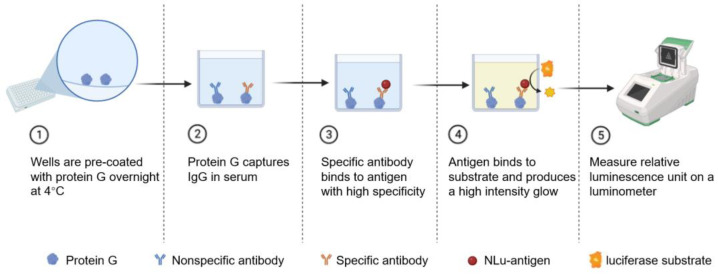
Schematic protocol for the luciferase immunosorbent assay (LISA).

As shown in Figure 1, white flat-bottomed high adsorption microtiter plates (Sangon Biotech, Shanghai, China) were coated with 5 μg/mL of protein G (Genscript) in 0.01 M PBS and incubated overnight at 4 °C. The plates were then washed three times with 0.01 M PBS containing 0.05% Tween 20 (PBST) and blocked with 5% skimmed milk for 1 h at 37 °C. PBST was used to wash the plates three times after the blocking step, and excess liquid was tapped dry. Afterward, serum samples (1:100 dilution in 2% skimmed milk) were added to each well and incubated at 37 °C for 1 h. Subsequently, the plate was washed three times, and the cell lysate (2% skimmed milk diluted to 3 μg/mL) was added to 50 μL of each well and incubated at 37 °C for 45 min. Following incubation, the wells were washed with PBST five times and dried. Finally, 50 μL of luciferase substrate (Promega, Madison, WI, USA) was added per well according to the instructions. The relative luminescence unit (RLU) was measured within 2 h using a Spark™ multimode microplate reader (TECAN, Männedorf, Switzerland). All samples were measured in at least three replicate wells. The cut-off value was determined as the mean +2 SD of the negative control group.

### 2.7. Development of CCHFV ELISA Based on Different NP Fusion Proteins

A commercial mouse Crimean–Congo hemorrhagic fever virus IgG antibody (CCHFV IgG) ELISA kit (Alpha Diagnostic International, San Antonio, TX, USA) was purchased as a control to verify the accuracy of the LISA method. The experimental process was performed in strict accordance with the manufacturer’s instructions. Briefly, all reagents were brought to room temperature (18–30 °C) for at least 30 min before use. First, 100 μL of each calibrator, samples, blanks, and controls were added to the pre-determined wells, the plates were gently tapped to mix the reagents, and the wells were washed four times after 60 min of incubation and patted dry. Then, 100 μL of diluted anti-mouse IgG HRP was added to each well. The wells were incubated for 30 min and washed 5 times. Next, 100 μL of TMB substrate was added to each well and incubated in the dark for 15 min before adding 100 μL of stop solution to each well. The absorbance of the whole plate was read at 450 nm using a single wavelength within 30 min after stop solution addition. The OD at 630 nm was subtracted to normalize the well background.

### 2.8. Statistical Analysis

Data are presented as the mean ± standard deviation (SD), and differences between groups were analyzed using appropriate statistical tests. A *p*-value of 0.05 was considered statistically significant. Statistical analyses were performed using GraphPad Prism 10.1 and Origin 2024.

## 3. Results

### 3.1. Expression of CCHFV NP Antigens Fused with Nano-Luciferase

**Figure 2:**
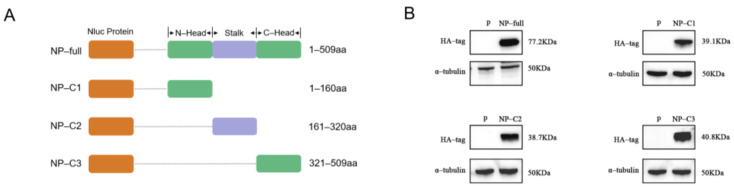
Design and expression of the NLuc-nucleoprotein fusion antigen. (**A**) Schematic design of the antigen detection fragments. The NP sequence was fused to the end of the NanoLuc sequence and then cloned into pNLF1-N to construct the recombinant plasmid; (**B**) recombinant plasmids were validated by Western blotting. Anti-HA tag antibodies were used to detect the four fusion proteins, NP-full, NP-C1, NP-C2 and NP-C3. α-tubulin was used as an internal control. *p*: empty plasmid pNLF1-N.

To establish a new approach for CCHFV detection, we constructed four luciferase expression plasmids containing either full-length CCHFV NP, the N-terminal head domain, the C-terminal head domain, or the stalk domain (Figure 2A). These plasmids were confirmed by restriction endonuclease reactions and gel electrophoresis. The NLuc-nucleoprotein fusion protein, expressed by mammalian 293T cells, was detected using Western blot (Figure 2B). These results confirmed the successful generation of the recombinant proteins for CCHFV-LISA.

### 3.2. Establishment of the LISA for the Detection of CCHFV IgG Antibody

Due to the high biocontainment requirements for CCHFV manipulation and the scarcity of samples, positive serum samples were acquired from antigen-immunized mice to support the development of CCHFV-LISA.

**Figure 3:**
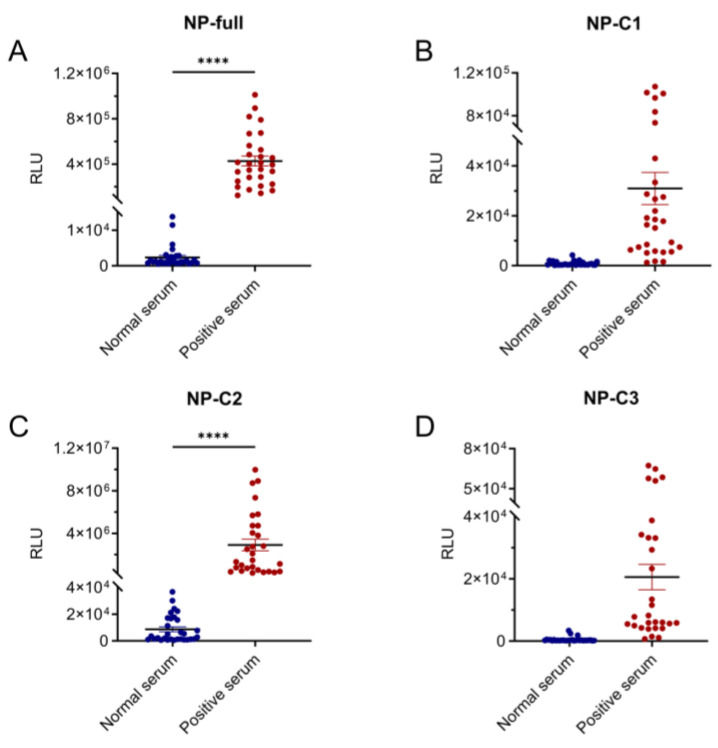
Number of positive detected of four recombinant detection fragments. Serum samples from healthy mice (“normal serum”) and CCHFV antigen-immunized mice (“positive serum”) were assessed for the relative luminescence unit (RLU) of anti-CCHFV IgG antibodies by NP-full LISA (**A**), NP-C1 LISA (**B**), NP-C2 LISA (**C**) and NP-C3 LISA (**D**). The serum dilution ratio was 1:100 and ****, *p* < 0.0001.

Based on the four recombinant antigen detection fragments, LISA was performed on the collected serum samples for sensitivity analysis. The positivity cut-off value was defined as the mean +2 SD of the normal serum RLU, and any sample with an RLU higher than the cut-off value was considered positive for CCHFV infection. The full-length fragments (NP-full) and NP-C2 fragments were able to detect all 29 positive serum samples, and the positive RLU was significantly higher than that of the negative control (*p* < 0.0001, Figure 3A,C). However, the NP-C1 and NP-C3 fragments could only detect 26 positive serum samples (Figure 3B,D). Therefore, NP-full and NP-C2 recombinant fragments were more effective in detecting mouse CCHFV IgG.

### 3.3. Optimal Structural Domain for CCHFV IgG Antibody Detection

**Figure 4:**
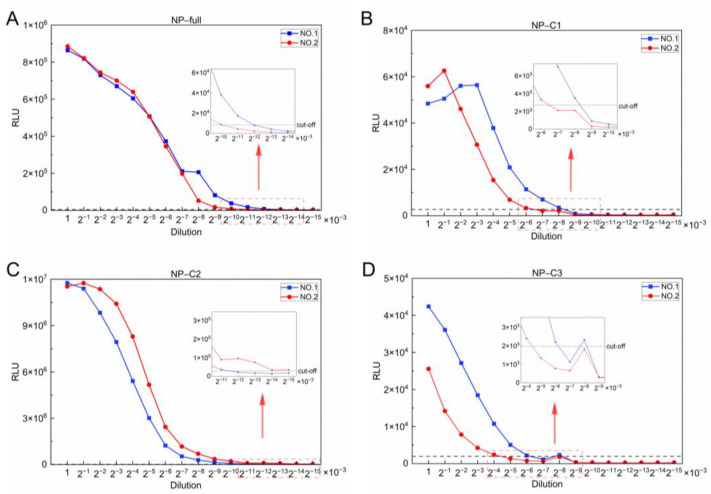
Sensitivity analysis of four recombinant detection fragments after gradient dilutions. Two positive mouse serum samples were randomly selected for serial dilution and RLU was determined using NP-full (**A**), NP-C1 (**B**), NP-C2 (**C**) and NP-C3 (**D**) detection fragments.

To further explore the optimal detection domain of the antigen, we performed gradient dilutions of two positive sera with a maximum dilution of 1:2^15^ × 10^3^. The lower limit of detection (LOD) of the fragments NP-C1 and NP-C3 was 1:2^8^ × 10^3^ (Figure 4B,D). By contrast, the well-performing fragment NP-C2 was able to detect IgG antibodies in positive samples at a dilution of 1:2^15^ × 10^3^ (Figure 4A), while NP-full LISA could not make this distinction at a dilution of 1:2^12^ × 10^3^ (Figure 4C). These results demonstrate that the NP-C2 LISA was at least eight times more sensitive than the NP-full LISA.

### 3.4. Comparison of the Novel LISA with the Commercial ELISA for Detecting Anti-CCHFV IgG

**Figure 5:**
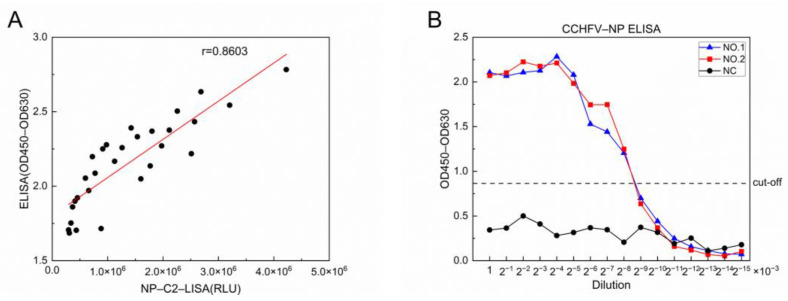
Comparison of the novel LISA with the commercial ELISA for detecting anti-CCHFV IgG. (**A**) Correlation between LISA and ELISA based on 29 positive samples. The RLU of the NP-C2 LISA is plotted against the absorbance of the ELISA (*p* < 0.0001). The serum dilution ratio was 1:1000. (**B**) Sensitivity analysis of ELISA. Measurements were carried out using a gradient dilution of serum, and the positivity cut-off value for positive results is indicated by the dashed line.

To evaluate the detection efficiency of the NP-C2 LISA in comparison to a commercial NP-based ELISA, we performed a correlation analysis. A strong correlation was observed between the two methods, with *Pearson’s* correlation coefficients of 0.8603 (Figure 5A).

Next, we utilized the ELISA to detect the gradient-diluted positive serum. The lower limit of detection for the ELISA was found to be in the range of 1:2^8^ × 10^3^–1:2^9^ × 10^3^ (Figure 5B). Notably, the ELISA for detecting CCHFV-infected IgG antibody positive sera dilution range was 50–2^8^ × 10^3^ times, and the sample dilution range for LISA was 100–2^15^ × 10^3^ times. This indicates that the lower limit of detection of the LISA was approximately 128-fold higher than that of the ELISA.

### 3.5. Repeatability and Cross-Reactivity of CCHFV-LISA

**Figure 6:**
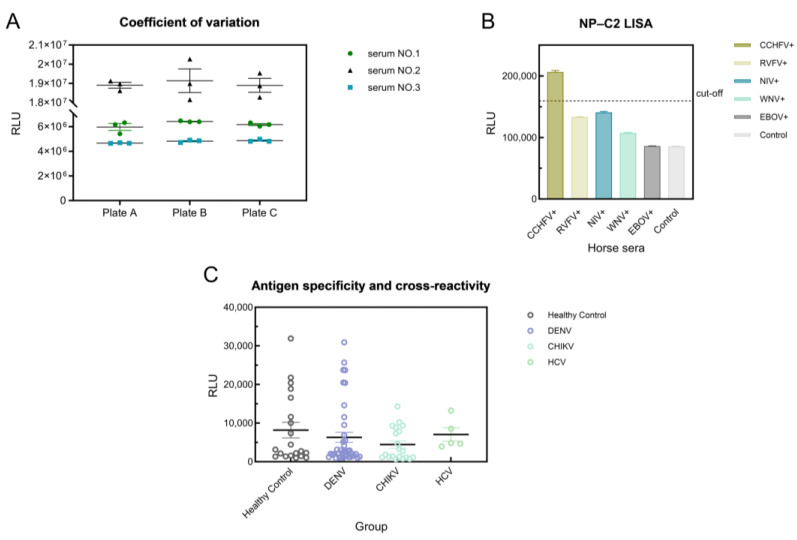
Repeatability and cross-reactivity of CCHFV-LISA. (**A**) Repeatability of IgG antibody detection by CCHFV LISA. (**B**) Identification of reaction specificity of NLu-NP-C2 protein among horse sera after infection with CCHFV, RVFV, NIV and EBOV. (**C**) Cross-reactivity of the NP-C2-LISA using positive sera from patients with DENV, CHIKV and HCV, and sera from healthy individuals served as controls.

To validate the detection efficiency for practical application, we assessed the repeatability and cross-reactivity of the CCHFV-LISA. In the repeatability experiment, three samples were used to determine the intra-assay and inter-assay CV values of the CCHFV-LISA and were found to be 1.66–8.50% and 0.62–8.13%, respectively (Figure 6A). These values are less than 10.0%, indicating satisfactory intra-assay and inter-assay repeatability.

To determine the cross-reactivity, we tested positive serum from a horse infected with CCHFV, RVFV, NIV, and EBOV and used negative sera to derive a threshold of 159,443. Only the CCHFV-infected horse serum was positive, with an RLU value of 206,218. By contrast, all other samples were negative, indicating no cross-reactivity with the CCHFV-LISA (Figure 6B). In addition, the cross-reactivity of CCHFV-LISA was further tested using a total of 85 convalescent-phase serum samples from humans, including 40 DENV-positive samples, 20 CHIKV-positive samples and 5 HCV-positive samples, with a cut-off value of 26,295 determined using 20 healthy individuals’ serum samples. No significant difference was observed among these groups (*p* > 0.1) (Figure 6C), demonstrating that the CCHFV-LISA did not detect IgG antibodies from the sera of patients with Chikungunya, dengue fever, or hepatitis C. Taken together, the NP-C2 fragment corresponding to the stalk structure exhibited good specificity.

## 4. Discussion

CCHFV is one of the most dangerous human pathogens and a potential bioterrorism agent, posing a serious threat to biosafety and public health [29,30]. As new or re-emerging infectious diseases, primarily of animal origin, become increasingly prevalent [31], CCHF, the most widespread tick-borne virus globally, remains a significant concern due to its enzootic tick–vertebrate–tick cycle [32,33]. Unfortunately, the lack of effective vaccines or specific drugs against CCHF places a significant health and economic burden [1,34]. Therefore, the development of new antibody detection methods for source tracing in natural foci is urgently needed. This study presents a new CCHFV serological assay for the rapid and sensitive detection of anti-CCHFV IgG antibodies.

NP is known to be the most conserved structural protein of CCHFV, exhibiting high antigenic similarity across genotypes and geographic locations [35]. This makes it an ideal candidate antigen for serological assays, as a specific genotype of NP can potentially apply to other genotypes. In addition, NP is the major antigen expressed after infection and is an ideal candidate antigen for the detection of viral antibodies [21]. Hence, NP is a rational target antigen for diagnostic and surveillance work, as well as for vaccine and drug development studies.

Although NP has been expressed in several expression systems, including baculoviruses and prokaryotes, its costly production and instability have limited its use in serodiagnostics [36,37,38]. The LISA developed in this study offers a solution using cell lysates for detection, eliminating the need for purification and post-expression modification. While viral isolation remains the gold standard for CCHF diagnosis, it requires high-containment biosafety level four facilities. To mitigate these risks, we employed a genetic engineering technique and eukaryotic expression system to produce antigen detection fragments. This approach not only preserved the natural epitopes of the CCHF virus but also eliminated the need for viral culture, significantly reducing the complexity and potential hazards associated with test antigen preparation. To assess the sensitivity and reproducibility of our newly developed CCHFV-LISA, we evaluated it using sera from mice. All 29 positive samples were accurately detected without a single false-positive sample. In addition, the coefficients of variation of inter- and intra-plate RLU of the same batch were lower than 10%. These results collectively demonstrate the efficiency and reproducibility of CCHFV-LISA.

We further compared the efficacy of CCHFV-LISA to a commercially available NP-based ELISA kit. The results showed a strong correlation between the two assays (*p* < 0.0001). In addition, we found that CCHF-LISA exhibited a significantly LOD for anti-CCHFV antibodies compared with the commercial ELISA. Furthermore, the LISA allows for the detection of CCHFV antibodies with minimal amounts of serum samples and detection antigens, meeting the requirements for testing. The CCHFV-LISA was still able to identify the positive samples even at the dilution of 2^15^ × 10^3^. Notably, the LOD of CCHFV-LISA is at least 128 times higher than that of commercial test kits, allowing for the earlier detection of IgG antibodies in patients. Unfortunately, the ELISA kit used in this study was only for mice; in the future, ELISA kits for a wider range of species should be used to compare the specificity of the two assays.

The identification of dominant antigenic epitopes is vital for the development of novel diagnostic tools. A previous report by Burt et al. found that the removal of the hydrophobic C-terminus of the NP did not affect the ability of ELISA to detect IgG antibodies in human serum. The region from amino acids 123–396 comprises a highly antigenic region of NP that can be used in the development of antibody detection assays [38]. Of the three structural domains of the NP, only the stalk domain detected all of the positive samples. Consistent with findings reported by Burt, the C-terminus of the NP had little effect on the ability to detect IgG antibodies in human serum. Considering the full-length NP also detected all positive samples, we further compared the detection sensitivity by gradient dilution. The NP-C2 fragment demonstrated good sensitivity by detecting positive samples even at higher dilutions, which was at least eight times more sensitive than the NP-full LISA. Similar to the region (123–396 aa) proposed by Burt [38], the stalk domain (161–320 aa) was further determined as the dominant antigenic region of NP for the detection of CCHFV IgG. However, IgG antibodies could not fully bind to the full-length NP, which may be related to the fact that the RBD is hidden after the protein is folded and expressed to form a spatial structure [26].

Cross-species serological testing facilitates the monitoring and prevention of CCHF transmission, as CCHFV can spread through multiple pathways, including from ticks to animals, from animals to humans, and between humans [39]. In contrast to common ELISA methods that require enzyme-linked species-specific antibodies, LISA offers a more versatile approach for tracking samples from various animals and vectors. In this study, CCHFV-positive horse sera and clinical serum samples from other viral infections with clinical signs similar to those of CCHFV infection were acquired for cross-species testing to verify cross-reactivity. The results showed that the NLu-NP-C2 fusion protein had a strong reaction specificity with CCHFV serum. Furthermore, all samples of CHIKV, DENV, and HCV tested negative, further confirming the high specificity of CCHFV-LISA.

Although obtaining positive serum samples from naturally infected individuals remains challenging for a comprehensive evaluation, we will continue our efforts to source samples from more species to validate the detection efficiency of CCHFV-LISA. Serum samples from other Bunyaviruses will also be sought to further analyze cross-reactivity with other viruses. In addition, LISA may have the potential to help detect other more zoonotic disease viruses, such as severe fever with thrombocytopenia syndrome virus, which is currently widespread in East and Southeast Asia [40]. Moreover, given the recent discovery of the Wetland virus, a closely related orthonairovirus to the CCHFV genome discovered in China [41], LISA could also be adapted as a rapid serological diagnostic tool for this emerging pathogen.

In conclusion, the CCHFV-LISA established in this study, based on the NP protein, is highly sensitive, cost-effective, and easy to use. It would be more suitable for on-site detection in large-scale epidemiological investigations and monitoring of CCHFV transmission, helping to mitigate its epidemic potential.

## Figures and Tables

**Table 1:** Recombinant antigen primer DNA sequence.

Amplified Gene	Primer Name	Primer Sequence	Fragment Size	Restriction Site
NP-C1	C1-F	GGCGCGATCGCTTCCGAATTCGCCACCATGCTCAAA	563	EcoRI
	C1-R	CAGCCAACTCAGCAAGCGGCCGCTTAAGCGTAATCTGGTACGTCGTATGGGTAATTGACACGGAAACC		NotI
NP-C2	C2-F	GGCGCGATCGCTTCCGAATTCGCCAACACAGCAGCT	554	EcoRI
	C2-R	CAGCCAACTCAGCAAGCGGCCGCTTAAGCGTAATCTGGTACGTCGTATGGGTATGCGCTTTGTGCACG		NotI
NP-C3	C3-F	GGCGCGATCGCTTCCGAATTCCAGATTGACACTGCT	614	EcoRI
	C3-R	CAGCCAACTCAGCAAGCGGCCGCTTAAGCGTAATCTGG		NotI

F, forward; R, reverse.

## Data Availability

Data are contained within the article.
